# Polar GaN Surfaces under Gallium Rich Conditions: Revised Thermodynamic Insights from Ab Initio Calculations

**DOI:** 10.3390/ma16175982

**Published:** 2023-08-31

**Authors:** Pawel Kempisty, Karol Kawka, Akira Kusaba, Yoshihiro Kangawa

**Affiliations:** 1 Institute of High Pressure Physics, Polish Academy of Sciences, Sokolowska 29/37, 01-142 Warsaw, Poland; 2 Research Institute for Applied Mechanics, Kyushu University, Fukuoka 816-8580, Japan

**Keywords:** density functional theory (DFT), phonon calculations, vibrational properties, GaN polar surfaces, surface thermodynamics, crystal growth, molecular beam epitaxy (MBE)

## Abstract

This paper presents an improved theoretical view of ab initio thermodynamics for polar GaN surfaces under gallium-rich conditions. The study uses density functional theory (DFT) calculations to systematically investigate the adsorption of gallium atoms on GaN polar surfaces, starting from the clean surface and progressing to the metallic multilayer. First principles phonon calculations are performed to determine vibrational free energies. Changes in the chemical potential of gallium adatoms are determined as a function of temperature and surface coverage. Three distinct ranges of Ga coverage with very low, medium, and high chemical potential are observed on the GaN(000-1) surface, while only two ranges with medium and high chemical potential are observed on the GaN(000-1) surface. The analysis confirms that a monolayer of Ga adatoms on the GaN(000-1) surface is highly stable over a wide range of temperatures. For a second adlayer at higher temperatures, it is energetically more favorable to form liquid droplets than a uniform crystalline adlayer. The second Ga layer on the GaN(0001) surface shows pseudo-crystalline properties even at a relatively high temperature. These results provide a better thermodynamic description of the surface state under conditions typical for molecular beam epitaxy and offer an interpretation of the observed growth window.

## 1. Introduction

Molecular Beam Epitaxy (MBE) is one of the growth methods of high-quality gallium nitride semiconductor structures used for light emitting diodes, lasers, solar cells, and high-frequency and power electronics. This technique operates at a relatively low growth temperature and under excess metal conditions [1]. Controlling the MBE growth of GaN is quite a complex process depending on many factors, mainly the species fluxes used and the substrate orientation and temperature. Typically, growth along the *c*-direction is achieved using the GaN(0001) surface, and more rarely the GaN(000-1) surface is used. Depending on the Ga-flux conditions and the substrate temperature, Ga-droplet, intermediate, and N-stable growth regimes are observed [2]. In practice, a narrow window of optimal step-flow mode growth in metal-rich conditions for plasma-assisted MBE (PAMBE) in the temperature range from 700 to 750 °C has been found [3]. Below this range, the formation of disturbing gallium droplets is observed, while above this range, increased GaN decomposition begins [2,3,4,5]. It is commonly believed that under optimal conditions, a very thin layer of pseudo-liquid metal (1–2 monolayers) remains on the growth surface. This model is derived from ab initio calculations and has been supported by experimental observations [6,7,8,9]. The Ga bilayer model explains very well the observation that in the Ga-rich smooth growth regime, the activation energy for Ga desorption from the growth surface is very close to the activation energy for evaporation of metallic gallium [10]. Based on quantitative in situ quadrupole mass spectrometry (QMS) measurements, the evolution of gallium coverage during PAMBE growth on the Ga-polar GaN surface was demonstrated [11,12,13,14]. The steady-state Ga-adlayer coverages increased continuously with the incoming Ga flux, reaching up to 2.5 monolayers before reaching the critical Ga flux for Ga-droplet formation. These dynamic studies generally confirm the results of previous static first principles total energy calculations, which predicted the stability of several key gallium adsorbate phases on the GaN(0001) surface. However, this consistency was qualitative and limited. These calculations considered only a limited number of surface reconstructions, such as Ga${}_{ad}$(2 × 2), Ga(1 × 1), and pseudo-(1 × 1) contracted Ga-bilayer.

While first principles calculations are used to study the properties of materials at the atomic and molecular levels, they do have important limitations. The core of this method lies in solving the Schrödinger equation for a many-body system. Naturally, the wave function describing a polyatomic system has numerous degrees of freedom related to both the number and type of particles present. This approach is highly computationally complex and usually requires the use of massive supercomputers. As a result, the size of the systems that can be analyzed is limited. Density Functional Theory (DFT) is one of the faster ab initio methods, which instead of the many-body wave function, uses the one-body electron density as the fundamental variable. This method facilitates the direct determination of the electron density and ground-state energy of the system through the principle of variation, applied to the energy as a functional of this density. The foundations of the DFT method are the Hohenberg–Kohn and Kohn–Sham theorems [15,16]. In principle, DFT is an almost parameterless method, relying solely on the definition of the chemical species of atoms. However, specific functionals are employed to parameterize the correlation and exchange interactions among electrons, and these functionals ultimately govern the accuracy of calculations [17]. DFT calculations are currently a standard and valuable research tool that allows for the calculation of the properties of atoms, molecules, and solids. Since DFT operates on systems in the ground state, the energy parameters of systems that would actually be at absolute zero temperature are determined. Thus, to achieve quantitative consistency, it is necessary to extend models to include high-temperature phenomena and entropic contributions.

Recently, Inatomi and Kangawa included an entropic factor in their analysis of adatom stability on polar GaN surfaces under MBE conditions [18]. This led to improved surface phase diagrams for the structures with the highest probability of appearance, which were in good agreement with experimental observations. However, the spatial resolution of their model was constrained by employing a small 2 × 2 slab with only four surface lattice sites. Generally, in previous DFT calculations for GaN surfaces, atomic systems typically did not exceed several tens of atoms and a few surface nodes.

In the present research, we conducted ab initio calculations to determine the energetic and thermal properties of polar GaN surfaces covered with Ga. These calculations were based on larger systems (over 200 atoms) and utilized a formalism that incorporates vibrational entropy [19]. Our aim is to better understand the thermodynamics associated with Ga adsorbates on polar GaN surfaces in the context of the phenomena observed during GaN growth by MBE method.

## 2. Calculation Methods

The quantum-mechanical calculations that underlie the present theoretical investigation were performed based on the density functional theory (DFT). The exchange-correlation energy functional was parametrized with the Perdew–Burke–Ernzerhof potential, in the form of a Generalized Gradient Approximation (GGA), using the Jellium Surface (Js), Jellium Response (Jr), and Lieb–Oxford bound criteria [20,21]. We employed the SIESTA package, which implements the pseudopotential method and numerical atomic orbitals as the set basis [22,23]. Pseudopotentials were generated with 2s^2^, 2p^3^ valence electrons for nitrogen and 3d^10^, 4s^2^, 4p^1^ for gallium. The basis size for both nitrogen and gallium atoms included triple zeta (TZ) functions for the *s* and *p* orbitals, and double zeta (DZ) for the *d* orbitals. The pseudopotential cutoff radii and the range of basis functions were optimized to achieve a balance between computation costs and the accuracy of the parameters for GaN, metallic Ga, and the N_2_ molecule. The obtained lattice constants of bulk GaN were a = b = 3.214 Å and c = 5.233 Å, which agree with experimentally determined values of a = b = 3.189 Å and c = 5.185 Å [24]. The dissociation energy of the N_2_ molecule was 9.93 eV, the cohesive energy of metallic Ga was 2.86 eV/atom, and the enthalpy of GaN formation was −1.31 eV, as calculated from the DFT total energy only.

The performed DFT calculations consisted of two main steps. In the first stage, the geometry of the systems (polar GaN surfaces with a double layer of metallic gallium adatoms) was optimized and the total energy was determined. In the second stage, phonon calculations were performed to determine the basic thermodynamic parameters of the system. Based on the obtained data, a thermodynamic analysis was performed to understand the behavior of the system at different temperatures.

### 2.1. Computational Model

The polar GaN surfaces were simulated using a slab model based on a supercell with a size of $2\sqrt{3}\times 2\sqrt{3}\times 4$ GaN unit cells, resulting in a slab with 12 surface sites and a thickness of eight Ga-N layers in the *c*-direction. To saturate the broken bonds on the opposite side of the slab, artificial pseudohydrogen atoms with atomic numbers of 0.75 and 1.25 were used for the nitrogen and gallium surfaces, respectively [25]. Gallium adatoms were placed systematically on the selected surface (N- or Ga-polar) until a double metallic layer was formed. In total, the system used for the calculations contained between 204 to 228 atoms, depending on the thickness of the adsorbate layer. A fixed cell size of 52.33 Å was used in the *c*-direction, resulting in a vacuum size of approximately 27 Å for the thickest adsorbate layer. Due to the asymmetric slab, a dipole correction was included [26]. The finite 3D mesh for real space calculations had a resolution of about 0.09 Å and was generated using a plane wave cutoff energy equivalent to 410 Ry.

Structural relaxation was performed by the Fast Inertial Relaxation Engine (FIRE) algorithm [27]. The positions of all atoms were relaxed until each atomic force dropped below $2\times 10^{-4}$ eV/Å. The convergence criteria for the Self Consistent Field (SCF) cycle in a single FIRE step were total energy fluctuation below $10^{-5}$ eV and a density matrix (DM) tolerance of $10^{-4}$ (maximum difference between the output and the input on each element of the DM in a SCF cycle). In the SCF cycle, a 2×2×1 *k*-point grid was used for Brillouin zone sampling. It was verified that in comparison to the 3×3×1*k*-grid, the differences in total energy did not exceed 0.5 meV/atom, while a 2.4-fold reduction in computation time almost proportional to the reduction in number of k-points was obtained.

Then, based on optimized structures, phonon calculations were performed using the simplest direct (frozen phonon) approach, which is based on the finite displacement method and harmonic approximation. In this method, phonons are determined from an interatomic force matrix calculated using an optimized supercell with atoms tilted from their equilibrium positions one at a time. More detailed information on this calculation technique can be found elsewhere [28,29,30]. Due to the high computational cost, with scales with the number of atoms times six steps, the procedure was only performed for a fragment of the slab containing the surface and its adjacent area for efficiency reasons. Specifically, the atoms of the gallium adsorbate and the three subsurface GaN layers were displaced by 0.011 Å from their optimal positions in each direction (−x, +x, −y, +y, −z, +z). Technically, the Vibra program included in the Siesta package was used to calculate the phonons. Phonon frequencies were collected over an 8×8×1 mesh in the wave–vector space. These spectra were used to determine the basic thermodynamic parameters of the system, including the zero-point vibration energy, thermal energy, specific heat capacity, vibrational entropy, and vibrational free energy. We deduced that for the double layer of metallic gallium on the GaN surface, the pseudo-crystal model is good enough. However, for thicker layers, the phonon calculations become less reliable because the Ga adatoms may approach the pseudo-liquid regime at a finite temperature. This means that at higher temperatures, the gallium layer may behave more like a liquid than a solid, which can affect its thermodynamic properties and behavior.

### 2.2. Thermodynamic Model

In simplified ab initio surface thermodynamics, the Gibbs energy is approximated using only enthalpy (*H*), which in turn is estimated by the total DFT energy of the system ($G\approx H\approx E^{DFT}$). However, this approach is not highly accurate as it neglects the contribution of entropy (*S*) and thermal effects. To improve the accuracy of the model, a full expression G(T)=H(T)−TS(T) that includes these contributions should be used. In the present study, it is assumed that the pressure-induced effects and thermal expansion of the crystals can be neglected. Therefore, the free energy can be expressed using the following equation that includes the total DFT energy, zero-point vibrational energy, thermal energy, vibrational entropy, and configurational entropy:

(1) $$\begin{array}{cc} \; & G^{slab}\left(T\right)=H\left(T\right)-TS\left(T\right)\\ \; & =E^{DFT}+E^{ZP}+E^{vib}\left(T\right)-{TS}^{vib}\left(T\right)-{TS}^{conf}\\ \; & =E^{DFT}+F^{vib}\left(T\right)-{TS}^{conf} \end{array}$$

The vibrational contribution to free energy can be determined from the phonon frequencies ω:

(2) $$F^{vib}\left(T\right)=\frac{1}{2}\sum_{i,q}\hbar \omega_{i}\left(q\right)+k_{B}T\sum_{i,q}\ln\left[1-\exp(-\frac{\hbar \omega_{i}\left(q\right)}{k_{B}T})\right]$$

where *ℏ* is the reduced Planck constant, $k_{B}$ is the Boltzmann constant, and the indices *i* and *q* denote the summation of all phonon modes at all sampling points in the wave vector space. The first term on the right is the zero-point vibrational energy.

In the grand canonical ensemble, which describes systems with a variable number of particles and allows for energy and particle exchange with a reservoir, the chemical potential (μ) is defined as the derivative of the Gibbs free energy with respect to the number of particles.

(3) $$\mu ={\left(\frac{\partial G}{\partial n}\right)}_{T}$$

Thus, by performing calculations for a series of slabs with a variable number of Ga adatoms, the chemical potential of the adsorbed atoms can be determined. By inserting Equation (1) into definition (3), the $\mu_{Ga}$ can be decomposed into ingredients:

(4) $$\mu_{Ga}\left(T\right)=\mu_{Ga}^{DFT}+\mu_{Ga}^{vib}\left(T\right)+\mu_{Ga}^{conf}\left(T\right)$$

This work focuses on determining the first two components: DFT and vibrational, as they are time-consuming tasks that require significant computational resources. The configurational contribution can be estimated separately using, for example, a simple ideal 2D lattice gas or ideal 2D gas approximation [31]. In the simplest case, considering only filled and empty nodes on the surface, the chemical potential coming from the configuration term can be defined as follows:

(5) $$\mu_{Ga}^{conf}\left(T\right)=k_{B}T\ln\frac{\theta }{1-\theta }$$

where θ is the coverage factor expressed as the ratio of the filled sites to all surface sites. This approach is good enough for cases with well-defined adsorption sites and in the temperature range where $k_{B}T$ is much lower than the diffusion activation energy.

When the surface is in contact with the vapor phase, the condition of equality of chemical potentials leads to:

(6) $$\begin{array}{cc} \mu_{Ga}^{surf} & =\mu_{Ga}^{vap}\\ \mu_{Ga}^{DFT}+\mu_{Ga}^{vib}\left(T\right)+k_{B}T\ln\frac{\theta }{1-\theta } & =\mu_{Ga_{vap}}^{\circ }\left(T\right)+k_{B}T\ln\frac{p^{eq}}{p^{\circ }} \end{array}$$

where $\mu_{Ga_{vap}}^{\circ }\left(T\right)$ is the temperature-dependent chemical potential of monoatomic gallium ideal gas at a pressure of $p^{\circ }$ = 1 bar and $p^{eq}$ is the equilibrium vapor pressure. The transformed Equation (7) finds the pressure needed to keep a surface in a certain state:

(7) $$p^{eq}=p^{\circ }\frac{\theta }{1-\theta }\exp\left(\frac{\mu_{Ga}^{DFT}+\mu_{Ga}^{vib}\left(T\right)-\mu_{Ga_{vap}}^{\circ }\left(T\right)}{k_{B}T}\right)$$

However, in the MBE method, particle flux (ϕ) is much more often used to describe growth conditions rather than pressure. Then it is possible to make a simple change of variables to calculate the equilibrium flux using the dependency:

(8) $$\phi =\frac{p}{\sqrt{2\pi m_{Ga}k_{B}T}}$$

Unfortunately, in the case of MBE processes, the situation is a bit more complicated because there is a one-dimensional gas moving in a given direction inside the chamber. Nevertheless, there are some methods to estimate the chemical potential of the particles from the effusion cells [32]. A precise description of the MBE growth of GaN would also require knowledge of the nitrogen flux, which co-determines the supersaturation above the surface and determines the minimum gallium flux required for the thermodynamic stability of GaN. Nevertheless, we are able to estimate the thermodynamic properties of metallic layers forming on the GaN surface.

## 3. Results and Discussion

### 3.1. Covering the Polar GaN Surface by Gallium Atoms

**Figure 1:**
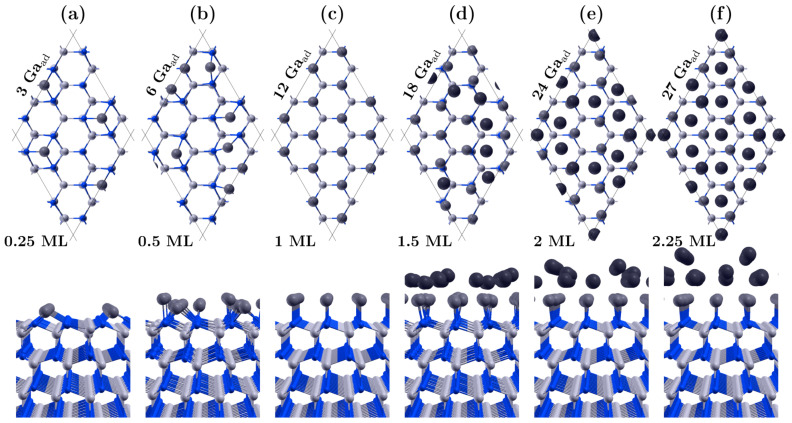
Atomic arrangements of some characteristic configurations of the gallium adsorbate on GaN(000-1) surface; nitrogen atoms are blue spheres, gallium are light gray spheres, and gallium adatoms in the first and second layers are marked with gray and dark gray spheres, respectively. The number of surface nodes in the GaN supercell is 12, and a notation of 1 ML = 12 adatoms has been employed. The subfigures present both top and side views of the GaN(000-1) surface, showing various numbers of Ga adatoms: (**a**) 3 Ga-0.25 ML, (**b**) 6 Ga-0.5 ML, (**c**) 12 Ga-1.0 ML, (**d**) 18 Ga-1.5 ML, (**e**) 24 Ga-2.0 ML, and (**f**) 27 Ga-2.25 ML.

**Figure 2:**
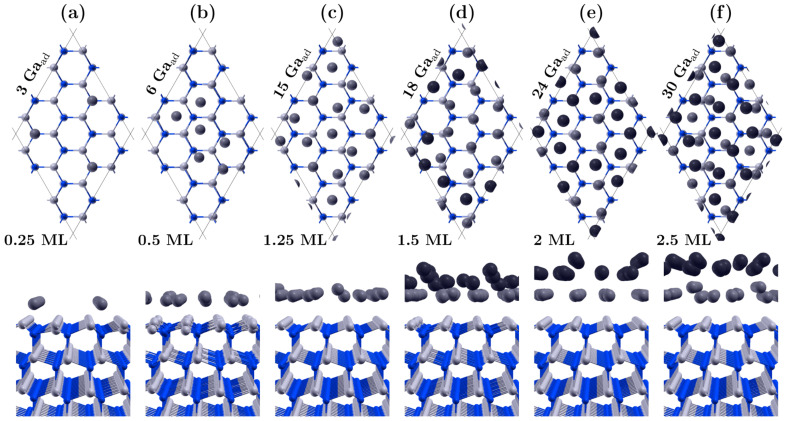
Atomic arrangements of some characteristic configurations of the gallium adsorbate on GaN(0001) surface; nitrogen atoms are blue spheres, gallium are light gray spheres, and gallium adatoms in the first and second layers are marked with gray and dark gray spheres, respectively. The number of surface nodes in the GaN supercell is 12, and a notation of 1 ML = 12 adatoms has been employed. The subfigures present both top and side views of the GaN(0001) surface, showing various numbers of Ga adatoms: (**a**) 3 Ga-0.25 ML, (**b**) 6 Ga-0.5 ML, (**c**) 15 Ga-1.25 ML, (**d**) 18 Ga-1.5 ML, (**e**) 24 Ga-2.0 ML, and (**f**) 30 Ga-2.5 ML.

The main DFT calculations involved systematically increasing the number of Ga adatoms on GaN polar surfaces, starting from clean surfaces and gradually obtaining a double metallic layer. For each coverage, several alternative lattice positions of adatoms on the surface were considered. Some characteristic configurations are shown in Figure 1 and Figure 2. At low Ga coverage, the energetically preferred positions were the triple bonded positions with high bonding energy: H3 for GaN(000-1) (Figure 1a) and T4 for GaN(0001) (Figure 2a), having 2×2 symmetry. This adsorption scenario proceeds to a coverage of 0.25 gallium monolayers (ML). At this point, we want to clarify that in the further considerations, we will use the notation in which 1 ML means the concentration of Ga adatoms equal to the concentration of gallium on the surface lattice of GaN (in our slab, 1 ML = 12 atoms).

For coverages above a 0.25 monolayer, mixed positions were observed, with the new adatoms mostly tending to attach in bridge positions via two bonds to surface atoms (see Figure 1b and Figure 2b). Notable differences were also observed between the two polar surfaces when covered with one monolayer of Ga. For the GaN(000-1) surface (Figure 1c), the ratio of Ga adatoms to surface nodes was precisely 1:1, and the adatoms occupied regular on-top positions above the nitrogen atoms, maintaining the order of the GaN lattice. However, such regularity was not observed for the GaN(0001) surface, where the 12 adatoms occupied different positions, mainly bridge and tilted on-top sites.

Furthermore, the packing density of Ga adatoms in the first full layer on the GaN(0001) surface could reach a value of 1.25, which translates to 15 Ga adatoms per 12 surface sites, as depicted in Figure 2c. The average bonding distance between two Ga adatoms in such a layer is approximately 2.6 Å, which is smaller than the lattice constant *a* of GaN. The metastable coverage range of 1.25 ML on the Ga-polar surface aligns with findings reported by Koblmuller et al. [13]. Our calculations reveal that as the coverage increases slightly beyond 1.25 ML, some adatoms move upwards to initiate the formation of the next layer. Energetically, it is more favorable to establish a lower density first layer with more adatoms in the second layer than to fully maximize the density of the lower layer with the minimal number of adatoms embedded in the upper layer. As an example, the lowest energy configuration with 16 Ga atoms (1.33 ML) consists of 3 adatoms in the top layer and 13 atoms in the bottom layer, rather than having 1 adatom in the top layer and 15 in the bottom layer. Ultimately, the density of the first layer may decrease from 1.25 ML to 1 ML if the total amount of gallium on the surface significantly exceeds 1.25 ML. This structural transition might seem a bit surprising but aligns with the unique properties of gallium, which crystallizes in complex structures [33].

The positions of atoms in the partially-filled second metallic layer do not show an evident continuation of either the GaN lattice or the first Ga monolayer. Instead, they adopt a more amorphous arrangement (see Figure 1d and Figure 2d). While it is possible to construct structures with high symmetry, they often have higher energy. As the coverage approaches 2 ML, a more regular lattice of adatoms with relatively lower energy emerges (see Figure 1e and Figure 2e). Moreover, the filling of the second layer can achieve a ratio of 1.25 ML. Thus, the fill factor for two full layers on the GaN(000-1) surface can attain a value of 2.25 ML, as shown in Figure 1f. On the (0001) surface, a slightly higher value is even possible, i.e., 2.5 ML, which is often cited in the experimental literature [11,12,13,14]. In this scenario, we observed that some adatoms can redistribute to the lower layer, allowing both layers to densify to 1.25 ML, as shown in Figure 2f.

The present section addressed the mechanical stability of adatoms on the surfaces. However, to achieve a deeper understanding of the involved phenomena, an analysis of electronic properties and thermodynamic factors is necessary.

### 3.2. Chemical Potential of Gallium Adatoms and Electronic Properties of Surfaces

**Figure 3:**
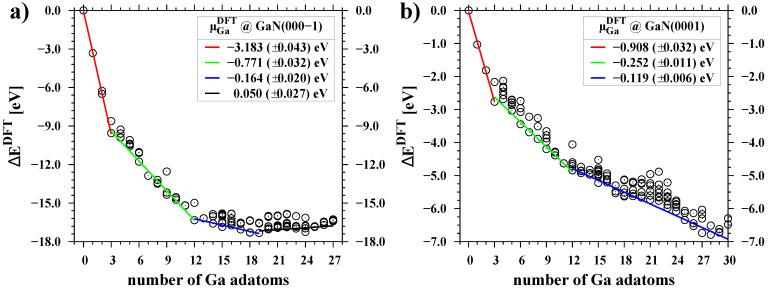
Change of DFT total energy as a function of the number of Ga adatoms on polar GaN surfaces, (**a**) GaN(000-1), (**b**) GaN(0001). The reference level is the sum of energies of the clean surface and the corresponding number of Ga atoms bound in the crystal. All data points (circles) represent energies obtained from DFT calculations for different adatom distributions on the surface. Linear relationships were fitted to the points with the lowest energies.

Figure 3 shows the DFT energies of the individually tested configurations plotted in a manner that enables us to determine the chemical potential of Ga adatoms according to Equation (3). This is achieved by calculating the derivative of the energy with respect to the number of particles. A linear relationship was fitted to the lowest energy points in several separate ranges of gallium coverages. The values of the determined potential $\mu_{Ga}^{DFT}$, expressed in relation to the binding energy of the gallium crystal, are listed in Table 1. The presented data demonstrate that Ga atoms adsorbed on a pure or nearly pure GaN(000-1) surface exhibit an extremely low chemical potential, approximately −3.18 eV below $\mu_{Ga}^{bulk}$. In contrast, for the GaN(0001) surface covered up to 0.25 ML, the value of $\mu_{Ga}^{DFT}$ is only −0.91 eV.

**Figure 4:**
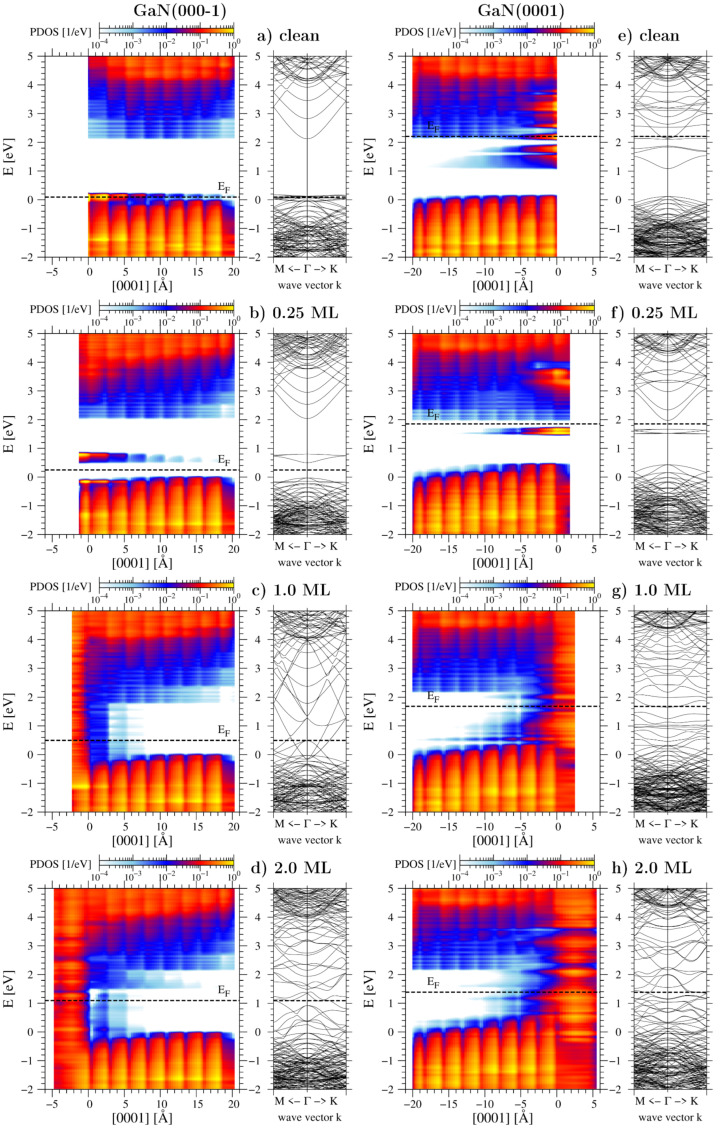
Spatial distribution of energy bands and band structure for GaN polar surfaces covered with varying degrees of gallium adatoms. The left column displays the results for the GaN(000-1) surface: clean (**a**) and covered with gallium adsorbate in the following amounts: (**b**) 0.25 ML, (**c**) 1.0 ML, and (**d**) 2.0 ML, respectively. The right column displays the results for the GaN(0001) surface: clean (**e**) and covered with gallium adsorbate in the amount of: (**f**) 0.25 ML, (**g**) 1.0 ML, (**h**) 2.0 ML, respectively. The position of both clean surfaces is indicated as 0 on the horizontal distance axis. The energy reference level is set to the valence band maximum away from the surface.

Previous DFT simulations have demonstrated that high adsorption energies in the low coverage range are attributed to the localized pinning of the Fermi level on partially filled surface states [34]. This phenomenon leads to the transfer of electrons from the aforementioned states to lower energy levels. For the GaN(000-1) surface, the surface states originating from unsaturated nitrogen bonds are degenerate with the valence band maximum, as illustrated in Figure 4a. When gallium attaches to this surface, a Ga-N covalent bond forms, causing the occupation of these low-energy states. Consequently, a significant energy gain of approximately 6 eV relative to a Ga atom in a vacuum occurs during adsorption within the coverage range of the 0.25 monolayer (ML). In the band gap, gallium-derived surface states also emerge, but they are positioned above the Fermi level (see Figure 4b). The situation is slightly different on the pure GaN(0001) surface. Here, the Fermi level is pinned to the gallium surface states and is positioned close to the conduction band (see Figure 4e). Following gallium adsorption, new occupied quantum states appear. However, due to their location within the energy gap (as shown in Figure 4f), these states are relatively high on the energy scale. Consequently, the energy gain during the adsorption process is approximately 2 eV smaller compared to the N-polar surface.

As the adsorption progresses from the 0.25 monolayer to the completion of the first monolayer on GaN(000-1), the value of $\mu_{Ga}^{DFT}$ remains stable at a moderate level of −0.77 eV. In contrast, for the GaN(0001) surface, this value is only −0.25 eV. With increasing density of adatoms, a greater number of gallium-derived states emerge within the band gap, causing a transition of both surfaces toward a metallic state. The band structures for coverages of 1 ML are presented in Figure 4c,g. It is evident that the Fermi level remains closer to the valence band for the GaN(000-1) surface, whereas it shifts nearer to the conduction band for the GaN(0001) surface. As a result, differences in adsorption energies and the chemical potentials of Ga adatoms within the coverage range of 0.25 ML to 1 ML persist between the two polar surfaces.

In the last range corresponding to the formation of the second adlayer on the surface, the Ga chemical potential is close to that of pure bulk gallium. For the (000-1) surface, two sub-ranges can be specified: $\mu_{Ga}^{DFT}$ = −0.25 eV for coverage in the range of 1–1.58 ML and 0.05 eV for 1.58–2.25 ML. On the other hand, the (0001) surface has a wider range of 1–2.5 ML, in which the Ga atoms exhibit a chemical potential of −0.12 eV. As shown in Figure 4d,h, both surfaces covered with 2 ML of Ga adatoms show subtle differences in their electronic properties. Particularly noteworthy is a minor opposing bend in the energy bands at the GaN-Ga interface, accompanied by a slight shift in the Fermi level. These distinctions appear to stem from the effects of the spontaneous polarization of GaN in the *c* direction. Generally, for thicker metal layers, the same chemical potential value close to $\mu_{Ga}^{bulk}$ is expected, regardless of the surface on which adsorption takes place. While we have not tested more than two adlayers, the screening effect is anticipated to eliminate the differences. However, some deviations in behavior from an ideal gallium crystal might occur due to the crystallographic structure changing as a consequence of lattice mismatch.

### 3.3. Temperature Dependence of Chemical Potential

The dependence of the chemical potential of Ga on the amount of adsorbate, as determined by changes in the total DFT energy, reflects only phenomena at very low temperatures. The next step in our investigation was to determine changes in the chemical potential as a function of temperature. Phonon calculations were performed for the previously considered systems and vibrational free energies were determined. However, for a small subset of the systems, the appearance of negative frequencies prevented the correct calculation of thermodynamic parameters. As a result, the number of configurations for which free energies could be determined was smaller than the number of configurations for which the DFT energy was calculated. For configurations that contained a large number of gallium adatoms, it was difficult to obtain a solution without negative modes, therefore the upper range of the analyzed coverages (above 2 ML) is examined with less precision. In the case of the GaN(0001) surface, we were unable to perform correct phonon calculations for 28–30 adatoms. A more advanced anharmonic approximation would probably help.

**Figure 5:**
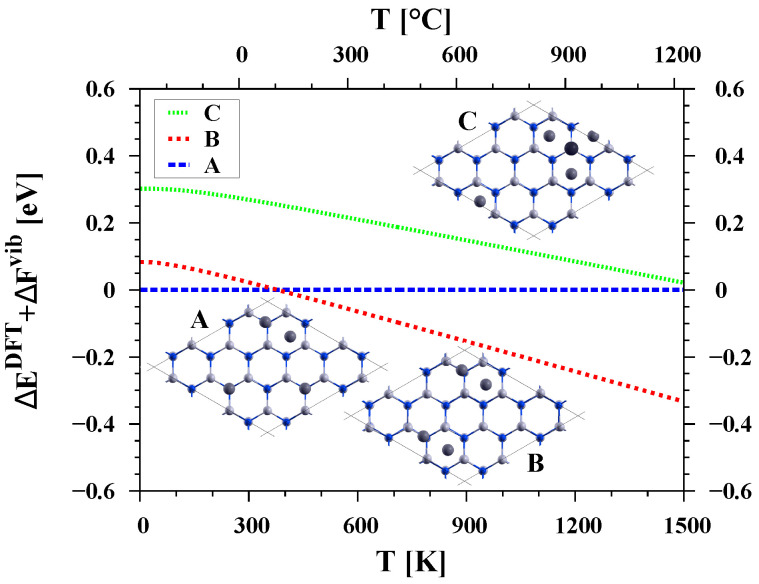
The difference in the free energy of the GaN(0001) surface with the same number of Ga adatoms (4) but different arrangements on the surface. Temperature dependence of configuration A was taken as reference.

In each of the systems with the same number of atoms but different arrangements, some of the free energy curves were parallel, while others intersected. This indicates that different configurations have the lowest energy at different temperatures, causing the preferred order of configurations to change depending on the temperature range. An example of such a relationship is shown in Figure 5, where at a temperature of about 380 K there is a transition between structure A (adsorbate in the form of single Ga atoms and Ga-Ga dimers) to structure B (only Ga-Ga dimers).

**Figure 6:**
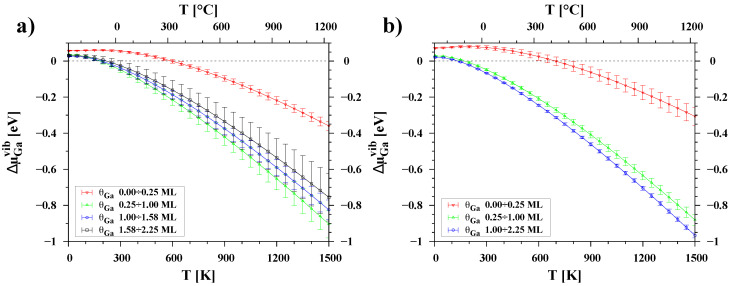
The changes in the vibrational chemical potential of Ga adatoms plotted as a function of temperature for (**a**) the GaN(000-1) surface and (**b**) the GaN(0001) surface. Each color of points represents a different range of surface coverage, $\theta_{Ga}$.

The chemical potential for multiple temperatures was determined using the same method as shown in Figure 3. However, this time, $\mu_{Ga}$ was calculated as the derivative of the sum of DFT energy and the free energy of vibrations over the number of adatoms. The calculation does not include the configurational component. The collected data points for individual temperatures were used to establish the dependence of $\mu_{Ga}\left(T\right)$. Figure 6 illustrates only the vibrational component of chemical potentials. At low temperatures, an increase in energy is observed due to the zero-point vibrational energy, whereas at higher temperatures, a decrease in energy is observed due to vibrational entropy. For both polar surfaces, the thermal changes of $\mu_{Ga}^{vib}$ are weaker at low coverages than at high coverages. The curves in Figure 6 for coverages up to 0.25 ML are clearly separated and have a lower slope, which is less than 0.4 eV in the range from 0 to 1500 K. In contrast, the other curves are almost parallel, and their thermal changes reach about 0.9 eV.

**Figure 7:**
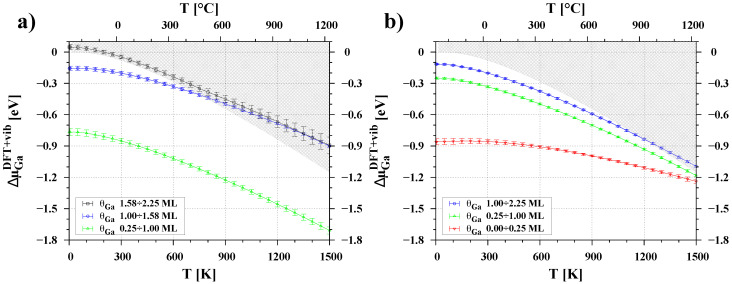
Temperature dependence of the chemical potential of Ga atoms adsorbed at the (**a**) GaN(000-1) surface and the (**b**) GaN(0001) surface. The vertical axis is the chemical potential relative to the $\mu_{Ga}^{bulk}$ at T = 0 K. Each color of points means a different surface coverage $\theta_{Ga}$. The gray hatched area indicates a chemical potential higher than the chemical potential of gallium in its crystalline and liquid states.

In the case of the GaN(000-1) surface (see Figure 7a), the chemical potential curves for the previously determined ranges of Ga coverage are well separated at low temperatures. However, the curve for the coverage below 0.25 ML is not visible on the graph as it lies far below the −3 eV vertical scale. Our analysis confirmed that even a monolayer of Ga adatoms is highly stable over a wide range of temperatures, with the chemical potential of the adsorbed Ga atoms remaining much lower (by at least 0.6 eV) than that of liquid gallium. As the amount of adsorbate increases (up to 1.58 ML) at a temperature of around 750 K, the chemical potentials of adatoms and liquid gallium become equal. This suggests that above this temperature it is energetically more favorable to form liquid droplets than a uniform pseudo-crystalline adlayer. The curve of $\mu_{Ga}$ determined for the second Ga adlayer on the surface coincides with the chemical potential of crystalline gallium and liquid gallium at low and medium temperatures, while at high temperatures it is clearly above $\mu_{Ga}^{liq}$. This confirms that the formation of a second regular layer of a typically crystalline nature is not possible because it enters the liquid regime.

In the case of the GaN(0001) surface (see Figure 7b), only coverages up to 0.25 ML of Ga can be considered highly stable at low and medium temperatures. The chemical potentials of adatoms with a coverage of 0.25 to 1 ML are below the chemical potential of liquid gallium, but the differences are relatively small: 0.2 eV at low temperatures and less than 0.1 eV at high temperatures. The $\mu_{Ga}$ curve for the second layer, i.e., from 1.0 ML to 2.25 ML, reaches the chemical potential of liquid gallium at temperatures around 1100 K. Nevertheless, the results indicate that a thicker layer of gallium adatoms can form on the GaN(0001) surface rather than on the GaN(000-1) surface. Differences in the slope of the curves for small and large Ga coverages result in similar chemical potentials of the adsorbate at high temperatures. This suggests that adatoms can move relatively easily between adsorption sites and form other configurations with similar free energies. This behavior is consistent with the results of ab initio molecular dynamics simulations by Bui et al. [35], who showed that the 2×2 Ga adatom reconstructions were stable at low temperature, while at 1300 K they formed dimer and trimer configurations, which are typical of higher coverages.

For broader usefulness, Table 2 also offers a straightforward parameterization of thermal changes in $\mu_{Ga}$ by fitting the following expression:

(9) $$\Delta \mu_{Ga}\left(T\right)=\mu_{Ga}^{0K}+a\;k_{B}T\ln\left[1-\;\exp\left(-\frac{b}{k_{B}T}\right)\right]$$

where Boltzmann constant $k_{B}$ is expressed in [eV/K] and the relevant fitting parameters are *a*, *b*, and $\mu_{Ga}^{0K}$ (chemical potential at 0 K). The parameters of $\mu_{Ga}^{0K}$ in Table 2 differ slightly from $\mu_{Ga}^{DFT}$ in Table 1 due to the inclusion of zero-point vibrational energy in the former.

The presented model of gallium adsorption is in good agreement with certain experimental observations. For instance, Held et al. demonstrated that at temperatures above 700 °C, the GaN(000-1) surface has at least two types of adsorption sites: the nitrided surface strongly binds Ga atoms, and when exposed to a sufficient amount of gallium, weak bonding sites emerge [36]. They also observed that the GaN(0001) surface adsorbs Ga only weakly. These behaviors correspond well to the chemical potential values shown in Figure 7. This picture is also in good agreement with the observations of Koblmuller et al., who stated that the Ga adlayer coverage differs substantially between the two surface polarities, being 1.1 monolayers on GaN(000-1) and 2.4 monolayers on GaN(0001) [11].

**Figure 8:**
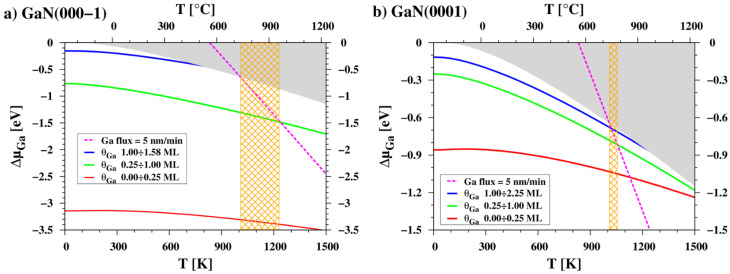
Temperature dependences of the chemical potential of the Ga adsorbate on (**a**) GaN(000-1), and (**b**) GaN(0001) surfaces, compared to the chemical potential of Ga atoms from the incoming molecular beam (magenta dashed line). The orange patterned rectangles indicate the temperature range in which a constant Ga flux ensures the formation of continuous adlayers on the surface. These temperatures prevent the formation of gallium droplets on the surface (left border of rectangle) and allow for the presence of at least 0.25 ML of gallium on the surface (right border of rectangle).

Based on our results, the growth window of GaN in MBE processes can also be explained. Figure 8 depicts the temperature dependence of the chemical potential of the Ga adsorbate on the GaN polar surfaces, compared to the chemical potential of Ga atoms in the vapor phase. For simplicity, we assume a typical constant flux of $36.5\times 10^{13}$ atoms/(s·cm^2^), corresponding to a GaN growth rate of 5 nm/min. Altering the flux value will result in a straight line with the same slope but shifted to the right or left, respectively, for higher or lower flux rates. Furthermore, when considering the influence of configurational entropy, the chemical potential curves of adatoms presented in Figure 7 and Figure 8 will gradually smear as the temperature increases, leading to a gradual change in surface composition.

Figure 8 also includes rectangles with orange crosshatch pattern, indicating a specific temperature range resulting from the intersection of the chemical potential curves of liquid gallium, adatoms, and vapor atoms. It is worth noting that these chemical potential curves represent the thermodynamic driving forces for the adsorption and desorption processes of the involved species. Within the indicated areas, it is possible to maintain a metallic adlayer on the surfaces. When temperatures fall below the lower limit, the chemical potential of Ga atoms in the beam becomes sufficiently high for droplets to form on the surface. The upper limit represents the temperature at which the surface can adsorb only 0.25 ML of Ga atoms. This range aligns effectively with MBE Ga-rich growth conditions, which are selected within the intermediate region between gallium droplet formation and significant gallium evaporation. This range is typically around 700–750 °C depending on the beam flux used. Figure 8 indicates that the optimal growth window for Ga-rich conditions should be wider on the N-side (about 210 °C) than on the Ga-side (about 45 °C) of GaN. This finding is in good agreement with experimental observations, where the growth of GaN on the (000-1) surface can be achieved at higher temperatures compared to GaN(0001) [37,38]. However, it is important to consider limitations arising from the thermal decomposition of GaN, a process heavily influenced by the nitrogen flux used.

## 4. Conclusions

In conclusion, this study employs ab initio thermodynamics to investigate the formation of gallium adlayers on polar GaN surfaces. Ordinary DFT calculations reveal that double layers of gallium can form on both polar surfaces of GaN. Incorporating phonon calculations enables the determination of changes in the free energy of the systems with respect to temperature, enhancing the quantitative consistency of the results. These thermal relationships introduce significant corrections to the chemical potential, refining the predictions based solely on DFT calculations at absolute zero temperature. The Ga adlayer coverage exhibits polarity-dependent differences. On the GaN(000-1) surface, a single gallium monolayer adopts a stable 1 × 1 reconstruction across a wide temperature range, while the second layer transforms into droplets at relatively low temperatures. Conversely, on the GaN(0001) surface, even at elevated temperatures, the presence of two continuous gallium adlayers is possible, with a total ratio of Ga atoms to GaN surface lattice sites potentially exceeding 2.25. This result agrees very well with previous experimental observations, indicating that in the case of the N-polar surface, achieving the Ga bilayer used as a surfactant in MBE growth on Ga-face GaN is not possible [4,11,12,13,14,38].

## Figures and Tables

**Table 1:** Chemical potential of Ga adatoms on GaN polar surfaces determined from DFT energies in four ranges of Ga coverage, according to the graphical representation in Figure 3. The reference level is the chemical potential of bulk gallium at T = 0 K taken from pure DFT energy.

Surface	Ga Coverage [ML]	Chemical Potential $\mu_{\mathrm{Ga}}^{\mathrm{DFT}}$ **[eV]**
GaN(000-1)	0.00–0.25	−3.183±0.043
	0.25–1.00	−0.771±0.032
	1.00–1.58	−0.164±0.020
	1.58–2.25	0.050±0.027
GaN(0001)	0.00–0.25	−0.908±0.032
	0.25–1.00	−0.252±0.011
	1.00–2.50	−0.119±0.006

**Table 2:** Parameters for fitting the temperature dependence of the chemical potential according to Equation (9).

Surface	Ga Coverage	Fitting Parameters		
	[ML]	$\mu_{\mathrm{Ga}}^{0K}$ **[eV]**	*a*	*b* [eV]
GaN(000-1)	0.00–0.25	−3.137	3.455	0.07304
	0.25–1.00	−0.768	2.811	0.01013
	1.00–1.58	−0.157	3.020	0.02087
	1.58–2.25	0.061	1.784	0.00204
GaN(0001)	0.00–0.25	−0.851	3.167	0.06327
	0.25–1.00	−0.256	2.924	0.01168
	1.00–2.25	−0.119	3.001	0.01088

## Data Availability

The data from DFT calculations are available for download from RepOD (Repository for Open Data) [39].
